# A Treatise on Syphilis

**Published:** 1830-04-01

**Authors:** 


### XLIX.

A TREATISE ON SYPHILIS, &c. By JOHN BACOT, Surgeon to the St. George's and St. James's Dispensary, &c. 8vo, pp. 280. London, 1829.

IT is not from a mean opinion of this volume that we have not hitherto reviewed it fully, or that we now merely content ourselves with a brief notice of it in our Periscope. The fact is, that having been previously published in the form of lectures in our valued contemporary, the Medical Gazette, it does not admit of any formal consideration in this Journal. We are loth, however, to pass over so deserving a work in silence, for we believe that if the precepts contained in it were engrafted in the minds of the younger members of the profession, and if they would but follow the practice recommended, the public eye might be spared the horror of many of those shocking objects, the victims of syphilis and mercury, that at present meet it in our marts and thoroughfares. There is not a disease in the black catalogue of human ills in which judicious practice may accomplish more good, or injudicious practice effect more evil, than in the dreaded and frequently dreadful lues. Not one in a hundred of those unfortunate beings that throng our alms' houses, and are sometimes seen in the mansions of the wealthy; wretches like Shakspeare's octogenarian, sans eyes, sans ears, sans teeth, sans every thing; not one in a hundred, we say, of those miserable creatures would be reduced to so loathsome a pass, did their medical attendant's information direct, or their own prudence allow, the proper measures to be steadily pursued. But this is a subject on which declamation would be idle, and we therefore hasten to give a specimen of the work before us. The subject we shall chuse is gonorrhœal rheumatism, on which our author's remarks though brief, are pithy and to the purpose.

"The next affection which I shall mention as a consequence of gonorrhœa is rheumatism; that is, pain and swelling of the knees and ankles especially. This is the most usual form which the complaint assumes, though in a few very rare instances the symptoms have been more general, the pain more acute, and the general disturbance of the system more severe. These diseases are scarcely mentioned by any writer upon venereal complaints, at which Swediaur expresses his astonishment; though, in fact, what he has said upon this subject is very unsatisfactory, and proves that it was but imperfectly known even to him; it has not, however, escaped the penetration of Mr. Brodie. Here, again, we are told that a suppression of the gonorrhœal discharge is the cause of the attack; but in the cases which have fallen under my own observation, this must be understood in a very qualified sense. I think it may be fairly said, that neither the affection of the joints, nor the more general rheumatism, come on until the gonorrhœa is upon the decline; and occasionally it has appeared to have succeeded to a sudden cessation of the discharge, following the use of cubebs or copaiba, in large doses; so that those medicines have not escaped the imputation of having been the remote causes of the attack. The subject is too little understood, and the examples of the disease too unfrequent, to permit me to indulge in theoretical views. All I can with confidence assert is, that an attack of pain, and enlargement of the joints of the knees and ankles, sometimes take place suddenly towards the termination of a gonorrhœa. The subjects of these attacks are usually young men of strumous habits, of florid complexions, and not particularly robust. There is often much puffiness and tenderness of the ankles, especially towards evening; the skin is not externally red; and the pain is not very much augmented by gentle pressure; the pulse is usually more frequent than in a state of health; the stomach sympathizes also in the attack; the appetite declines, or fails altogether; and now and then it happens that all these symptoms are suddenly relieved by an eruption of papulæ, in clusters; or sometimes by pustules, in very minute patches. When these appear, not only are the pains relieved, but the constitutional symptoms also yield; and the eruption, after some days, sometimes, indeed, not for some weeks, grows paler, and a desquamation succeeds, leaving a slightly discoloured state of the skin, which, however, gradually wears itself out. This is the progress of the symptoms when left to themselves; but medicine can do much to relieve them, and to facilitate and hasten their course. In the first attack of pain and swelling of the joints, rest, and confinement to bed, together with the employment of local or general blood-letting, will be necessary; though the use of the lancet is, I think, upon the whole, much to be preferred to the application of leeches; but the bleeding should not be carried to any extent. This should be accompanied with the exhibition of saline antimonial medicines, combined with the compound powder of ipecacuanha, in doses of five or six grains, with an interval of four or five hours between each; or what sometimes answers still better, the vinum colchici, in such doses as will produce some effect upon the stomach and bowels. For this purpose, one drachm of the wine may be given as a single dose, mixed with magnesia and camphorated mixture, and a very sudden remission of the pain is frequently the consequence; or, if preferred, the same remedy may be given in more divided doses, from twenty to twenty-five minims every five or six hours. When, by either or all of these means, the pains are relieved, and the pulse returns to its healthy standard, frictions to the limbs, either of camphoretted spirits, or with the flesh-brush, and the internal use of the compound decoction of sarsaparilla, will tend to restore the tone and vigour of the system. If the joints continue swollen and stiff, the warm salt-water bath may be used three times in the week, and a moderate share of exercise permitted, provided the weather admits of it.

"In those cases where the affection of the joints is succeeded by eruptions of the papular or pustular forms, (sometimes, indeed, they are mingled together in the same individual), in addition to the sarsaparilla, small alterative doses of mercury may be conjoined. Of these the best form is, I believe, the compound calomel pill of the present pharmacopœia. Under its judicious and careful use the eruptions will fade away much more quickly, and the strength and health will be more speedily restored than by the mere vegetable remedy alone. It is not necessary, even in these cases, to carry the exhibition of mercury to the extent of salivation, though a slight tenderness of the gums is not by any means objectionable. One caution, however, is, I think, absolutely necessary; that is, never to persevere in the use of the mercury if it deranges the bowels, or appears to excite any disturbance in the system, denoted by acceleration of the pulse, restlessness, or disturbed sleep at night. Such is the plan of treatment which I should adopt in these affections; but when we have to encounter the more rare, but at the same time more formidable cases of general rheumatism, the mode of treatment must be assimilated to that which we should practise in cases unconnected with any gonorrhœal origin; that is, bleeding may occasionally be necessary. Antimonials or colchicum, with opium and the warm-bath, will be indicated according to the extent and severity of the symptoms; though in the convalescent state the sea-air and bathing are equally appropriate, and more necessary even than in the former instances.

"Among the medicines most efficacious in removing the chronic stage of this disease, bark and guaicum hold the first rank. The ammoniated tincture of guaicum is, indeed, in these instances, a most invaluable remedy, given in doses of from forty to sixty drops, in combination with the decoction of bark, two or three times in the day."

Mr. Bacot has once or twice found these rheumatic complaints dependent on an irritable state of the urethra, the consequence of long-continued or repeated discharge. Here a painful condition of the feet is often one of the most distressing symptoms. In such cases the cure cannot be expected until, by the employment of bougies, the urethra is restored to its natural state. Mr. Bacot pays a well-merited compliment to Mr. Brodie, for the accuracy of that distinguished surgeon's observations on this disease in his Treatise on Diseases of the Joints. Mr. Brodie, it is well known, thinks very highly of the powers of colchicum. Mr. Bacot agrees with Mr. Brodie in believing that the pains are aggravated by blistering the swollen joints, and he does not therefore recommend the measure.

In two cases Mr. Bacot has witnessed ulcerations of the soft palate, leading to disease of the ossa palati, consecutive on a virulent gonorrhœa, the discharge of which had apparently been cured about two months previously. The first stage of this secondary affection was an inflammatory flush of the whole palatine arch; a small pimple then formed and burst just where the velum pendulum palati begins; this spread rapidly until the ulceration assumed the size of a silver three-pence; and continued there with a sloughy bottom, and without much pain, but indisposed to heal by all the simple means employed for that purpose. The patient was of a very irritable strumous habit, and the first appearance of the disease was accompanied with much fever, which gave way to active purging and antimonials. Sarsaparilla was afterwards freely employed, but it was only when mercury was conjoined that a cure was effected. In one case the course appeared not to have been carried to a sufficient extent: the ulceration broke out afresh: disease of the superior maxillary bone ensued: exfoliation took place: and the patient finally recovered after a long course of mercury.

"These cases are, I conceive, highly interesting, because they are certainly proofs of affections of the throat and spongy bones, directly arising from gonorrhœa, and gonorrhœa only. They are rare, perhaps very rare occurrences, not sufficiently common to cause a revolution in our practice, but sufficiently important to call our attention to any similar affection which we must not reject as syphilitic, and withhold the exhibition of mercury merely because we can only trace gonorrhœa as a primary symptom. We must recollect how much is depending upon our coming to a right decision upon a question of such importance to the comfort and welfare of our patient, and not obstinately refuse a remedy which, judiciously managed, will undoubtedly lead to a successful issue, because the phenomena are not exactly in accordance with our preconceived notions. This is a subject to which my attention has lately been particularly called, and it stands in need of farther elucidation."

As far as the history afforded by a patient goes, we may say that we have on several occasions seen severe ulcerations of the throat succeeding gonorrhœa only. But the mischief of it is, that if the accounts of their complaints delivered by patients are frequently fallacious on other occasions, they are positively deceptive to a degree in venereal maladies. They cannot, or they will not, tell the truth, and the surgeon is but too often obliged to judge for himself, and decide upon a plan in direct opposition to what the "history" would point out. In the cases to which we alluded sarsaparilla was effectual, as it commonly is in these secondary sore throats, the compounds of the venereal and mercurial virus.

Here we must conclude, and we cordially recommend Mr. Bacot's book to those who wish to have a comprehensive view of what is generally known and practised by the best surgeons in respect to syphilis.

